# Emergence of Classical BSE Strain Properties during Serial Passages of H-BSE in Wild-Type Mice

**DOI:** 10.1371/journal.pone.0015839

**Published:** 2011-01-14

**Authors:** Thierry Baron, Johann Vulin, Anne-Gaëlle Biacabe, Latefa Lakhdar, Jérémy Verchere, Juan-Maria Torres, Anna Bencsik

**Affiliations:** 1 Agence Française de Sécurité Sanitaire des Aliments – Lyon, Lyon, France; 2 Istituto Nacional de Investigatcion y Tecnologia Agraria y Alimentaria, Madrid, Spain; Creighton University, United States of America

## Abstract

**Background:**

Two distinct forms of atypical spongiform encephalopathies (H-BSE and L-BSE) have recently been identified in cattle. Transmission studies in several wild-type or transgenic mouse models showed that these forms were associated with two distinct major strains of infectious agents, which also differed from the unique strain that had been isolated from cases of classical BSE during the food-borne epizootic disease.

**Methodology/Principal Findings:**

H-BSE was monitored during three serial passages in C57BL/6 mice. On second passage, most of the inoculated mice showed molecular features of the abnormal prion protein (PrP^d^) and brain lesions similar to those observed at first passage, but clearly distinct from those of classical BSE in this mouse model. These features were similarly maintained during a third passage. However, on second passage, some of the mice exhibited distinctly different molecular and lesion characteristics, reminiscent of classical BSE in C57Bl/6 mice. These similarities were confirmed on third passage from such mice, for which the same survival time was also observed as with classical BSE adapted to C57Bl/6 mice. Lymphotropism was rarely detected in mice with H-BSE features. In contrast, PrP^d^ was detectable, on third passage, in the spleens of most mice exhibiting classical BSE features, the pattern being indistinguishable from that found in C57Bl/6 mice infected with classical BSE.

**Conclusion/Significance:**

Our data demonstrate the emergence of a prion strain with features similar to classical BSE during serial passages of H-BSE in wild-type mice. Such findings might help to explain the origin of the classical BSE epizootic disease, which could have originated from a putatively sporadic form of BSE.

## Introduction

The unique features of the transmissible agent involved in the food-borne epizootic disease of bovine spongiform encephalopathy (BSE), namely the incubation periods of the disease, the distribution and features of the neuropathological lesions, as well as the molecular features of the disease-associated prion protein (PrP^d^), have been characterized following transmission studies in inbred wild-type mice [1]–[4]. These features, as assessed by transmission in mice, appeared to be remarkably stable, even following cross-species transmission from cattle to other species, particularly that which occurred under natural conditions in humans to produce the variant Creutzfeldt-Jakob disease (CJD), or in some animal species such as domestic cat or goat [5]–[9]. However, the origin of this transmissible agent remains a mystery, even though recycling of a scrapie agent from small ruminants has often been suspected [10], [11].

Recent studies have shown the existence of three different prion diseases in cattle, based notably on the molecular features of the protease-resistant prion protein (PrP^res^) identified by Western blot [12]–[15]. These bovine TSEs include (i) classical BSE (C-BSE), associated with the prion strain identified during the food-borne BSE epizootic disease in Europe since the 1980's, (ii) H-type BSE (H-BSE), which is an uncommon type originally described in France [13] and (iii) L-type BSE (L-BSE), also known as bovine amyloidotic spongiform encephalopathy (BASE), a rare form of BSE first identified in Italy [12]. H- and L-BSE differ notably from classical BSE by the respectively higher or lower apparent molecular mass of unglycosylated PrP^res^ observed in Western blot [15]. These two diseases have now been recognized in other European countries [15], Japan [16] and North America [17], [18], and are suspected to represent sporadic forms of prion diseases, as are most cases of Creutzfeldt-Jakob disease in humans [11], [19]. It is therefore probable that such cases of BSE existed before the onset of the classical BSE epizootic disease. It has also been hypothesized that food-borne transmission of L-BSE could have been at the origin of the several outbreaks of transmissible mink encephalopathy (TME) identified since 1947 in ranch-raised mink [20].

H-BSE and L-BSE have been shown to differ between each other and from classical BSE with respect to their incubation periods, vacuolar pathology in the brain, and biochemical properties of PrP^res^, following transmission in transgenic mice that express the bovine prion protein [21]–[25]. In addition, after first-passage transmission of H-BSE, C57Bl/6 wild-type mice showed different features from classical BSE, including the distinct molecular features that characterize the disease in cattle [26], [27]. In contrast, L-BSE apparently failed to transmit the disease to C57Bl/6 or SJL wild-type mice on first passage [24]. These data indicated that three distinct major strains of TSE agents were involved in the three phenotypes of BSE in cattle. However, transmission studies showed that L-BSE could acquire similar phenotypic traits to those of the classical BSE agent, during cross-species transmission in either inbred wild-type mouse lines [24] or in a transgenic mouse model (tg338) over-expressing ovine PrP [23]. This led to the hypothesis that conversion of the L-BSE agent, resulting from passage in an intermediate host, could explain the origin of classical BSE.

In this study, we demonstrate that although the distinct biochemical and histopathological features of H-BSE can be maintained for at least three passages in C57Bl/6 wild-type mice, the emergence of classical BSE properties may occur during serial passages in some of the animals.

## Results

**Figure 1:**
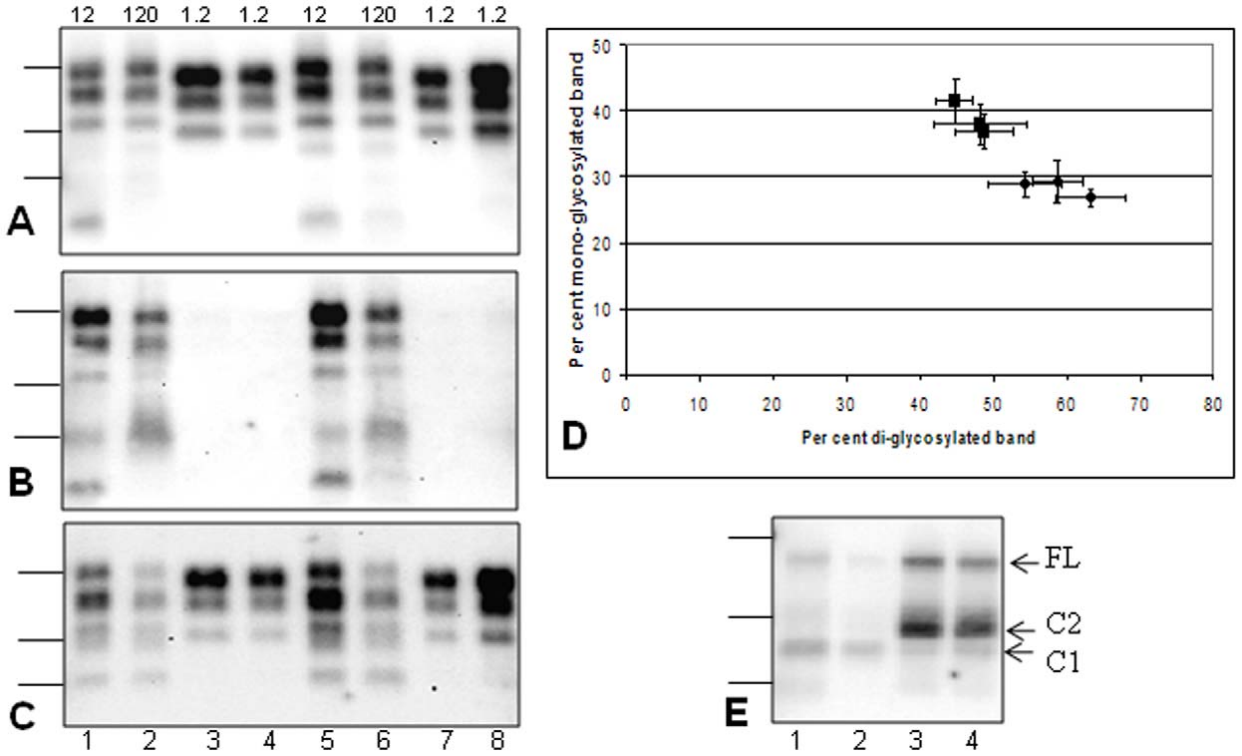
Western blot analyses of brain PrP^res^ from mice infected with H-BSE. Mice inoculated with with H-BSE (lanes 1–3 and 5–7 from isolates 01-2604 and 03-2095 respectively), at first (lanes 1, 5) or second passage (lanes 2–3 and 6–7), were compared to mice infected with classical BSE (lanes 4, 8) in panels A–C. At second passage, PrP^res^ exhibited either H-type features, from mice that died later (491 and 504 d.p.i. in lanes 2 and 6 respectively), or “C-BSE like” features (464 and 322 d.p.i. in lanes 3 and 7 respectively). The brain tissue equivalents loaded per lane are indicated (in tenth of mg). D - Glycoform proportions (means +/− standard deviations) of PrP^res^ from H-BSE (isolate 03-2095)-infected mice detected using Sha31 antibody. Mice exhibited PrP^res^ with a molecular mass either similar to that of H-BSE (full squares) or to classical BSE (full diamonds). E - PrP^d^ extracted in the absence of protease digestion and deglycosylated by PNGase treatment from H-BSE infected mice at first (lane 1) or second passage (lanes 2-3 from mice that died at 497 d.p.i. and 464 d.p.i. respectively), compared to a classical BSE control in lane 4 (0.3 mg brain tissue equivalent per lane). Full-length PrP^d^ (FL) and C1 and C2 fragments are indicated. Panels A and B were revealed by monoclonal antibodies Sha31 and 12B2 respectively and panels C and E by HRP-labelled SAF84 antibody. Bars to the left indicate the 29.0, 20.1 and 14.3 kDa marker positions.

Primary transmission of two H-BSE isolates (01-2604 and 03-2095) was previously reported in C57Bl/6 mice, which showed the same molecular features of the protease-resistant prion protein (PrP^res^) in Western blot as initially described in cattle. These features were a ∼1.5 kDa higher apparent molecular mass of the three PrP^res^ glycoforms compared to that found in mice infected with a classical BSE isolate, associated with strong labeling by the 12B2 antibody in H-BSE but not in classical BSE (Figure 1A and B, lanes 1 and 5)[27]. In addition, a C-terminally cleaved form of PrP^res^ (PrP^res^ #2), with an unglycosylated form migrating at ∼14 kDa, was identified by probing with C-terminal antibodies such as SAF84 monoclonal antibody (Figure 1C, lanes 1 and 5)[26] and PrP^d^ was revealed by immunohistochemistry solely as amyloid plaques [27].

### PrP^res^ molecular features in the brains of H-BSE serially passaged in C57Bl/6 mice

Transmission of the disease was then obtained on second passage of three different H-BSE isolates in C57Bl/6 mice with detection of the disease-associated prion protein PrP^d^ by Western blot and/or immunohistochemistry in most (37/41) of the mice inoculated with 1% brain homogenates, as shown in Table 1. The mean mice survival periods were 560, 560 and 453 days post-inoculation (d.p.i.) for these three H-BSE isolates i.e., 142 - 199 days less than for the primary transmissions.

**Table 1:** Bovine TSE transmission to C57Bl/6 mice. Survival periods of the animals and results of PrP^res^ detection and molecular analysis by Western blot.

		Survival periods			Survival period according to the	
Isolate	Nature	(mean +/- s.d., d.p.i.)^1^			molecular phenotype at 2^nd^ passage	
		1^st^ passage	2^nd^ passage	3^rd^ passage	H-type PrP^res^	“C-BSE like” PrP^res^
01-2604	H-BSE	702+/−117 (8/9)	560+/−84 (15/16)	ND	491–763 (11)^3^	464 (1)
03-1928	H-BSE	705+/−96 (6/10)	560+/−55 (13/15)	ND	469–654 (12)	(0)
		744+/−44 (8/13)	ND	ND		
03-2095	H-BSE	652+/−85 (10/10)	453+/−90 (9/10)	183+/−6 (15/16)(C)^2^	492–654 (5)	322–405 (4)
				721+/−121 (14/16 (H)^2^		
			362+/−104 (10/11)^4^	ND		253–427 (8)
01-2281	C-BSE	520+/−84 (10/16)	208+/−11 (10/20)	ND	ND	ND

1 : number of positive mice by Western blot and/or immunohistochemistry/number of mice examined.

2 : (C) or (H) indicates that the mice were inoculated with a mouse brain with C-type or H-type features at second passage.

3 : number of mice with the indicated PrP^res^ phenotype identified by Western blot.

4 : inoculation of a 10% brain homogenate instead of 1% homogenates in all the other second and third passage experiments.

ND: not done.

On this second passage in C57Bl/6 mice using 1% brain homogenates, similar molecular features to those described at first passage (H-type) were observed in mice (26/26) surviving after 469 d.p.i. (Table 1 and Figure 1). However, some mice (1 and 4 respectively) in two of the three experimental groups had shorter survival times (322-464 d.p.i.) and exhibited a strikingly different molecular pattern, with a lower (∼1.5 kDa) apparent molecular mass of the three PrP^res^ glycoforms (Figure 1A) and an absence of labeling by 12B2 antibody (Figure 1B). No PrP^res^ #2 signal could be detected using SAF84 antibody in these five mice (Figure 1C), contrary to the mice with H-type PrP^res^. In comparison to mice with H-type features, the proportions of diglycosylated PrP^res^ demonstrated by probing with Sha31 antibody, were also increased, (Figures 1A, D). The molecular features of PrP^res^ in these mice were thus indistinguishable from those of C57Bl/6 mice infected with classical BSE (“C-BSE like”).

From one of these isolates (03-2095), following inoculation of a 10% instead of a 1% brain homogenate, the mean survival period was reduced to 362 days (Table 1) and all the mice (8/8) examined by Western blot, that died between 253 and 427 d.p.i., showed “C-BSE like” features.

A third passage was also performed from this 03-2095 H-type isolate, using 1% mouse brain homogenates that exhibited either H-type or “C-BSE like” features. The resulting survival periods were strikingly different (721+/− 121 d.p.i. and 183+/− 6 d.p.i. respectively), and similar to those described on first passage of H-BSE [27] and after adaptation of classical BSE [28] respectively. All PrP^res^ positive mice (14/16) inoculated with H-type brain homogenate exhibited H-type PrP^res^ features, whereas the molecular features of all mice inoculated with the “C-BSE like” brain homogenate were indistinguishable from those of classical BSE.

It should be noted that the PrP^res^ levels in mice with “C-BSE like” features were much higher than in mice with H-type PrP^res^ and, in this regard, were comparable to mice infected with classical BSE, as shown by the different equivalent brain tissue masses that needed to be loaded to obtain equivalent PrP^res^ signals (Figures 1A and 1C). These differences in levels of the disease-associated PrP accumulating in the mouse brains were also demonstrated by analyzing brain homogenates in the absence of proteinase K (PK) treatment (Figure 1E). After loading similar quantities of brain equivalent tissues on each lane, cleaved PrP, corresponding to the C2 fragment, was only abundant in mice with “C-BSE like” features. In mice exhibiting H-type PrP^res^ (i.e., all mice on first passage and mice surviving longest on second passage), low levels of C2 fragment were detected in addition to full length PrP and the C1 fragment which were detected in all mice, including the non-inoculated ones. These data suggest that the differences in PrP^res^ loads between mice with H-type or “C-BSE like” features are not linked to different PK sensitivities, but rather to genuine differences in PrP^d^ accumulation.

### PrP^res^ molecular features in the spleen using H-BSE serially passaged in C57Bl/6 mice

**Figure 2:**
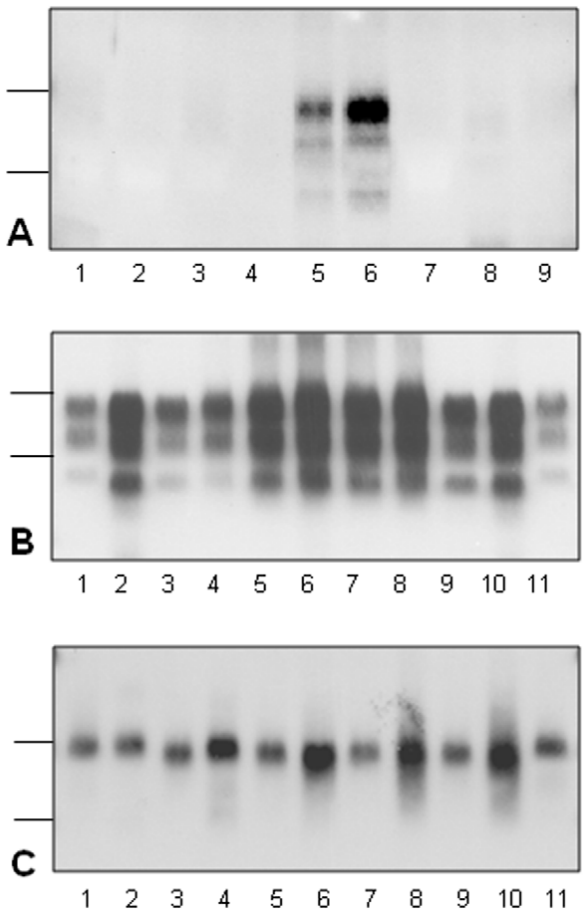
Western blot analyses of PrP^res^ in the spleens of mice infected with H-BSE. A - Detection of PrP^res^ from spleens of mice inoculated with H-BSE isolates 01-2604 (lanes 1–3) or 03-2095 (lanes 4–9) at first passage. B - Detection of PrP^res^ from spleens of mice inoculated with H-BSE at third passage in mice inoculated with a brain homogenate with “C-BSE like” PrP^res^ (lanes 4–8). PrP^res^ from the brain of one of these mice (lanes 3, 9) and from brains of C506M3 scrapie (lanes 1, 11) or classical BSE (lanes 2, 10) controls are shown for comparison. C - PrP^res^ from spleens of mice infected with H-BSE at first passage (lanes 2, 4)(isolates 03-1928 and 03-2095), second passage in a mouse with “C-BSE like” PrP^res^ (lane 6) and third passage from mice inoculated with a brain homogenate with “C-BSE like” PrP^res^ (lanes 8, 10). PrP^res^ from spleens of classical BSE (lanes 3, 5, 7, 9) and C506M3 (lanes 1, 11) controls are shown. Panels A and B were revealed by monoclonal antibody Sha31 and panel C by HRP-labelled SAF84 antibody Bars to the left indicate the 29.0 and 20.1 kDa marker positions in panels A and B or the 20.1 and 14.3 kDa marker positions in panels C and D.

PrP^res^ could not be detected by Western blot in spleens from any of the mice infected with the 01-2604 H-BSE isolate at either 1^st^ or 2^nd^ passage, but was detected in all mice infected with the classical BSE isolate at both passages (10/10 and 9/9 respectively)(Table 2). Low levels of PrP^res^ were detected after first passage in 2/20and 2/11 mice inoculated with the two other H-BSE isolates (Figure 2A). After PNGase deglycosylation, the molecular masses were higher (∼0.5 kDa difference) than in mice infected with classical BSE and comparable to those of mice infected with the C506M3 scrapie strain (Figure 2C). In contrast to classical BSE, PrP^res^ was also labeled by 12B2 antibody (data not shown). However, we were unable to clearly identify a C-terminal PrP^res^ product by probing with the SAF84 antibody, in contrast to our observations on brain tissue.

**Table 2:** Western blot detection of PrP^res^ into the spleens of C57Bl/6 mice during serial passage of H-BSE.

		Western blot detection of PrP^res^ in the spleen^1^		
Isolate	Nature	1^st^ passage	2^nd^ passage	3^rd^ passage
01-2604	H-BSE	0/9	0/14	ND
03-1928	H-BSE	0/10	0/14	ND
		2/10	ND	ND
03-2095	H-BSE	2/11	1/10	6/7 (C)^2^
				0/16 (H)^2^
01-2281	C-BSE	10/10	9/9	ND

1 : number of Western blot positive mice/number of mice examined.

2 : (C) or (H) indicates that the mice were inoculated with a mouse brain with C-type or H-type features at second passage.

ND: not done.

After second passage, PrP^res^ was detected in a single mouse inoculated with one of the three H-BSE isolates. This mouse also had the shortest survival period (322 d.p.i.) in the experimental group and showed “C-BSE like” PrP^res^ in the brain. The features of PrP^res^ in this mouse were similar to those of mice infected with classical BSE and migrated faster than the C506M3 scrapie control (Figure 2C). No PrP^res^ could be detected in the spleens of the four other mice with “C-BSE like” PrP^res^ in their brains. On third passage, PrP^res^ was readily detected in 6/7 mice inoculated with the brain homogenate associated with “C-BSE like” features (Figure 2B). PrP^res^ from the spleens of these mice migrated similarly to that of mice infected with classical BSE, as shown after PNGase deglycosylation (Figure 2C). In contrast, PrP^res^ could not be detected in the spleens of 16 mice inoculated with the H-type brain homogenate at this third passage.

### Neuropathological studies of H-BSE serially passaged in C57Bl/6 mice

**Figure 3:**
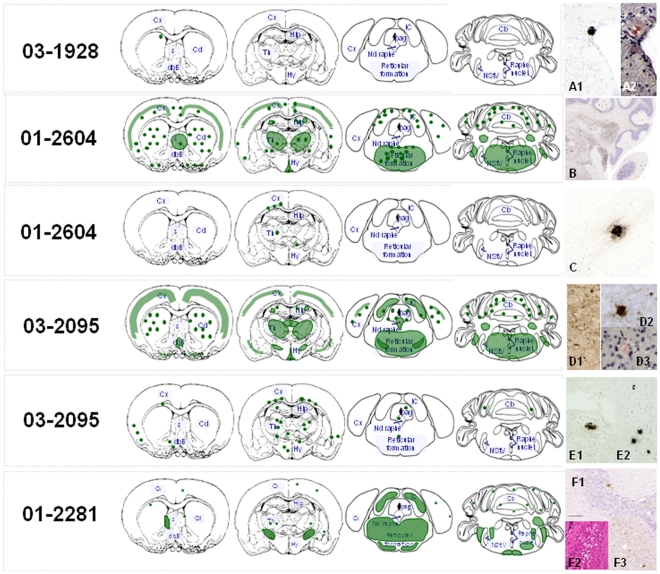
Histopathological analyses of mice infected with H-BSE at second passage. Brain distribution of the disease-associated prion protein (PrP^d^) observed in the brain of C57Bl/6 mice infected with 3 isolates of H-BSE, at second passage. The green color gives the schematic representation of PrP^d^, the dots symbolize the plaque type deposits. Plaques of amyloid nature as revealed by a Red Congo staining observed under polarized light (A2 & D3) and made of PrPd deposits (A1, D2, E1, E2) (black staining after IHC) were similarly detected in each H- BSE transmission studies and remarkably this amyloid type of PrP^d^ deposition was the predominant histopathological features typical of H-BSE. In two out of three second passage experiments, another sub-group of mice was clearly identified as showing a different PrP^d^ brain mapping. This sub-group showed much more brain areas accumulating PrP^d^ with a granular type of staining (B, D1), reminding most of the features seen in the case of the classical BSE features (F1, F3) of which also typical spongiform changes in the cochlear nucleus (F2).

Because of the unexpected sub-group occurrence, it was not possible to build strong brain lesion profiles with at least 5 to 6 mice for each sub-group (see supplemental data). Still the neuropathological analysis of the H-BSE second passage experiments based on PrP^d^ analysis offered an unequivocal evidence of the existence of two different sub-groups of mice in two out of three experiments. One subgroup showed the histopathological features seen in H-BSE at first passage, and especially a low amount of PrP^d^ which was exclusively detected, by Congo red staining, as amyloid plaques (Figure 3A1, A2, C, E1, E2). The other subgroup showed clearly distinct histopathological features. In particular, the PrP^d^ deposits were much more numerous and PrP^d^ brain mapping was similar in both experiments (Figure 3B and D1). Interestingly, the types of PrP^d^ deposition and PrP^d^ brain mapping were reminiscent of those described previously for classical BSE in C57Bl/6 mice [29].

**Figure 4:**
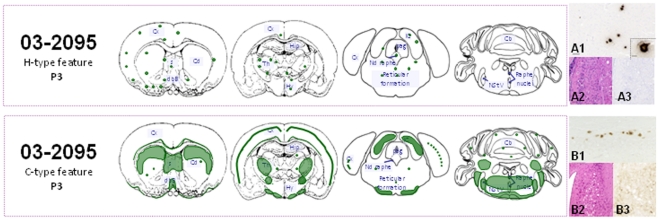
Histopathological analyses of mice infected with H-BSE at third passage. Brain distribution of the disease-associated prion protein (PrP^d^) observed in the brain of C57Bl/6 mice infected with H-BSE, at third passage from a mouse with H-type features (A) or with “C-BSE like” features (B). The green color gives the schematic representation of PrP^d^, the dots symbolize the plaque type deposits. In right panels, pictures of some characteristics PrP^d^ deposits for 3^rd^ passage of H-BSE from mouse with H-type features in thalamus as plaques solely (A1) or from mouse with C-type features in the cortex (B1). The cochlear nucleus showed spongiform lesions (B2 compared to A2) and granular PrP^d^ brain deposits (dark deposits of DAB intensified using NiCl2) (B3 compared to A3) in the C-type solely. Scale bars: 50 µm for all panels and 10 µm for the plaque focus in panel A1.

After a third passage, neuropathological analyses of mice inoculated with a mouse brain homogenate exhibiting H-type PrP^res^ revealed less vacuolization of the cerebellum and hypothalamus while the thalamus and septum presented the most severe vacuolization (Figure S1). Notably, the cochlear nuclei were completely unscathed (Figure 4A2). PrP^d^ brain mapping analyses revealed several characteristic features: i) PrP^d^ accumulated almost exclusively as plaques (Figure 4A1); ii) the brainstem, cerebellum and cochlear nucleus (Figure 4A3) did not show any PrP^d^ accumulation whereas the thalamus, midbrain and cortex presented PrP^d^ plaques (Figure 4A) and iii) the rostral region of the cortex was markedly more highly labeled.

Neuropathological analyses of mice, after third passage transmission from a mouse with “C-BSE like” PrP^res^, again indicated similarities with classical BSE as illustrated by the comparative PrP^d^ brain mapping (Figures 3 and 4B). Labeling of the cochlear nucleus and cerebellum was particularly marked and the PrP^d^ deposits in the latter showed the same aggregate morphology. Nevertheless, the cortex and thalamus were less strongly labeled in the classical BSE control and for unknown reasons labeling of the thalamic nuclei was not identical in the two groups.

## Discussion

Our study shows that after second passage transmission of H-BSE in C57Bl/6 mice, the features described after first passage transmission [27] were maintained in most of the mice. The observed mean survival periods were much longer than for classical BSE. The phenotypic features of the disease were (1) PrP^res^ of higher apparent molecular mass, associated with 12B2 labeling, indicating a different cleavage site of the protein (2) presence of an additional C-terminal PrP^res^ product (PrP^res^ #2) specifically detected with C-terminal antibodies (SAF84)[26] and (3) the deposition of PrP^d^, mainly in amyloid plaques, as detected by immunohistochemistry [27]. Different strain-specific PrP^d^ cleavages have also been shown between classical scrapie and BSE. This was demonstrated in sheep using immunohistochemical analyses of the brain [30] and, after transmission in an ovine transgenic mouse model, by Western blot after PrP^d^ extraction in the absence of any protease digestion [31]. In addition we show here that, in contrast with classical BSE, H-BSE is poorly lymphotropic in C57Bl/6 mice, including after serial passages.

Strikingly different phenotypic features were observed in a few mice, which also exhibited shorter survival periods, after this second passage of H-BSE in C57Bl/6 mice. These features included (1) a similar apparent molecular mass of PrP^res^ to that found in mice infected with classical BSE [32] and the absence of a C-terminal PrP^res^ product detected by SAF84 antibody [26] and (2) lesions of vacuolar degeneration and patterns of PrP^d^ deposition characteristic of classical BSE previously described in these mice [29], [33]. After a third passage, using mouse brains with “C-BSE like” features, the biochemical and histopathological similarities with classical BSE were confirmed, whereas the characteristics of H-BSE were maintained in mice inoculated with a brain homogenate containing H-type PrP^res^. In addition, most (6/7) of the mice inoculated with a brain homogenate with “C-BSE like” features were positive in the spleen and showed similar features to those found with classical BSE, in contrast with mice with H-type PrP^res^ at third passage.

Our data demonstrate the emergence of phenotypic features similar to those of classical BSE after cross-species transmission of an atypical form of BSE and are reminiscent of those previously described for the other form of atypical BSE, i.e., BASE or L-BSE [24]. L-BSE otherwise shows clearly distinct features from H-BSE, both in cattle and after transmission in different transgenic mouse models, thereby indicating that the two atypical forms of BSE involve two distinct major strains, both of which differ from the strain involved in classical BSE [12], [15], [21]–[23], [34]. It is thus intriguing that the two atypical forms are able to show similar phenotypic features to those of classical BSE after cross-species transmission in wild-type mice. The L-BSE study, which involved monitoring a single L-BSE isolate during two passages in C57Bl/6 and SJL wild-type mice and a single passage in RIII and VM mice, nevertheless differed from our study on several points. The mice were inoculated by both intra-cerebral and intra-peritoneal routes, from a bovine thalamus sample at first passage and from pools of brains from C57Bl/6 or SJL mice at second passage. No evidence of disease transmission was found on first passage [24], although trace amounts of PrP^res^ were reported in a single RIII mouse that, interestingly, showed biochemical properties identical to those of classical BSE-infected mice. The situation in our experiments was different since H-BSE was able to transmit the disease to C57Bl/6 mice after first passage, with accumulation of PrP^d^ in the brain. Mice with “C-BSE like” characteristics were identified at the second passage, that showed the shortest survival times (322-464 days post-inoculation (d.p.i.)), as compared with the survival times of 258-331 d.p.i. reported after second passage in C57Bl/6 mice, with a L-BSE isolate [24]. However, the other mice in our experiments developed H-BSE and exhibited longer survival periods (> 491 d.p.i.), and similar biochemical and histopathological features to those observed at first passage. The explanation of shortened survivals of mice at this second passage is unclear, but it does not seem to reflect an adaptation of the H-type BSE agent in C57Bl/6 mice since the survival of mice that showed H-type features at third passage was longer. Both Western blot and histopathological analyses demonstrated the maintenance of similar features (either H-type or “C-BSE” like”), after third passage, in all mice inoculated with either one of the two phenotypes. The above observations suggest the existence of a divergence phenomenon whereby the H-BSE properties are maintained in only some of the infected mice during serial passages, possibly as a result of the coexistence of both agents and competition between them. Further investigations are required to determine the level of interference between the two agents that are able to propagate in C57Bl/6 mice.

However, the emergence of phenotypic properties similar to classical BSE in some C57Bl/6 mice infected with H-BSE in our study was observed in only two out of three experiments involving the three isolates. Although the possibility of cross-contamination with classical BSE cannot be excluded unequivocally as classical BSE has been handled in our laboratories, the occurrence of cross-contamination is highly unlikely as we have implemented rigorous laboratory practices and there has been no evidence of cross- contamination in previous experiments that have been conducted in our laboratory. The possibility that the observations might be influenced by the precise neuro-anatomical origin of the inoculated bovine brain stem homogenate cannot be excluded. We can however hypothesize that classical BSE might represent a minor sub-population of the TSE agents present in atypical H-BSE which could be selected by serial passages in wild-type mice. Alternatively, a TSE strain similar to classical BSE might be produced *de novo* during cross-species transmission of H-BSE prions in wild-type mice. Such observations are not unprecedented and can be compared to the sudden and discontinuous changes observed in some scrapie strains with Class III stability, such as the 87A scrapie strain in C57BL mice. In some experiments, 40% and 100% of the mice inoculated with 1% and 10% wt/vol brain homogenates respectively showed much shorter incubation periods, at all stages between primary and seventh passage. Serial passages from such mice consistently led to isolation of a novel strain (7D) with stable incubation periods and distinct neuropathological features, indistinguishable from those of the ME7 scrapie strain [35]. These observations were interpreted as evidence of a mutation of the scrapie agent. Another example has been the identification of two distinct strains (HY and DY) from transmissible mink encephalopathy after serial passages in hamster, which was interpreted as selection of strains from a mixture [36]. Further studies of the possible effects of inocula concentrations and serial passages are thus required in the case of H-BSE, to better understand the similar “breakdown” phenomenon [35] that was observed in our study. Interestingly an experiment of a second passage of H-type BSE using a more highly concentrated (10%) brain homogenate, from a mouse brain that had already provided evidence of C-BSE emergence as 1% brain homogenate, favored the propagation of the “C-BSE like” agent.

Our study may help to clarify our understanding of the relationship existing between - most probably sporadic - forms of atypical BSE and the food-borne epizootic disease of classical BSE in cattle.

## Materials and Methods

### Ethics statement

Experiments were performed in the approved experimental facilities (A3) of the author's institution (n° A 69 387 081) with the approval of the Rhône-Alpes Ethical Committee for Animal Experiments (CREEA n°98) and following the guidelines of the French Ethical Committee (decree 87-848) and European Community Directive 86/609/EEC.

### Bovine TSE isolates

Cattle TSE isolates included 3 H-BSE isolates and 1 classical BSE isolate, based on the molecular analyses of PrP^res^ (Table 1)[13]. Features of the disease and PrP^res^ molecular features after a primary passage in C57Bl/6 mice have previously been described for two of the H-BSE and for the classical BSE isolate [26], [27].

### Transmission studies in mice

For serial passages, four-to-six weeks old female mice (15–20 animals per experimental group) were inoculated intra-cerebrally with 1% (wt/vol) brain homogenates in 5% glucose (20 µl per animal) from mice at 1^st^ or 2^nd^ passage. Mice were followed twice weekly and at the terminal stage of disease or end of life, brains were collected and either analysed, from frozen samples, by Western Blot, or from fixed samples in buffered 10% formalin, by histology and immunohistochemistry. Spleens were frozen for Western blot analysis.

### Western blot analyses of PrP^res^ and PrP^d^

The extraction methods used to identify and characterize the proteinase K (PK) resistant prion protein (PrP^res^) from mouse brains have been previously described [26], [37]. Briefly, PrP^res^ was obtained following treatment of mouse brain homogenates with PK (Roche)(10 µg/100 mg brain tissue for 1 h at 37°C) and concentration by ultra-centrifuging (100 000 rpm for 2 hours on a 10% sucrose cushion). Disease-associated PrP (PrP^d^) isolated from the brains of mice was also prepared for Western blot analyses in the absence of PK treatment, and was isolated as previously described for PrP^res^, but the PK digestion step was omitted [31]. For spleen samples PrP^res^ was extracted from the entire spleens by treating the spleen homogenates with collagenase (100 µg/100 mg spleen in a 1 ml total volume) and DNAse (64 µg/100 mg spleen in a 1 ml total volume) for 1 h at 37°C, then with PK (24 µg/100 mg spleen in a 1.2 ml volume) for 1 h at 37°C [38]. In some experiments deglycosylation was performed using PNGase F (kit P07043, BioLabs). Denatured samples of PrP^res^ in TD4215 buffer (4% sodium dodecyl sulfate, 2% β-mercaptoethanol, 192 mM glycine, 25 mM Tris, 5% sucrose)(1–2 µl) were mixed with denaturing buffer from the PNGase kit, G7 buffer, NP40 and PNGase according to the manufacturer's instructions. After incubation at 37°C for 1 h, samples were ready for Western blot analysis following appropriate dilution in TD4215 buffer.

After heat denaturation for 5 min at 100°C in TD4215 buffer, PrP was separated in 15% SDS-PAGE and electroblotted on to nitrocellulose membranes, then detected on the membrane using anti-PrP monoclonal antibodies. PrP^res^ or PrP^d^ were detected using the monoclonal antibodies anti-PrP Sha31 (1/10 from kit TeSeE sheep/goat Biorad), SAF84 (500 ng/ml)(SPI-Bio, France) or 12B2 (340 ng/ml) against the 144-WEDRYYRE-151, 163-RPVDQY-168 and 88-WGQGG-92 murine PrP sequences respectively. Peroxidase-labelled conjugate anti-mouse IgG (H+L)(1/2500 in PBST)(ref 1010-05)(Clinisciences, France) was used to detect Sha31 and 12B2 antibodies, whereas SAF84 was used as horseradish peroxidase antibody. Quantitative studies of PrP^res^ polypeptide molecular mass and glycoforms proportions were performed using Quantity One (Biorad) software analysis of chemiluminescent signals. Glycoforms ratios were expressed as mean percentages (+/− standard deviations) of the total signal for the three PrP^res^ glycoforms and the apparent molecular masses were evaluated by comparison of the positions of the PrP^res^ bands with a biotinylated marker (B2787, Sigma).

### Histopathological analyses

Mouse brains fixed in buffered 10% formalin solution were treated for 1 hour at room temperature (RT) with formic acid (98–100%) before embedding in paraffin blocks (Thermo Electron, Cergy-Pontoise, France). Tissue sections five micrometers thick were cut from paraffin blocks, placed on treated glass slides (Starfrost, Medite Histotechnic, Burgdorf, Germany) and dried overnight at 55°C. Once dewaxed, the slides were stained for either histopathological or immunohistochemical examination. Amyloid deposits were identified with a Congo red stain, and vacuolar lesions were observed on slides stained with hematoxylin-eosin (HE) according to Fraser's lesion profile analyses [39]. Lesion profiles were measured using a computer-assisted method [40]. For immunohistochemistry, brain slices were immunostained for the presence of disease-associated prion protein (PrP^d^) using 2 µg/ml of anti-PrP SAF84 monoclonal antibody (SPI Bio, France) [41]. Recently described pre-treatments designed to enhance PrP^d^ detection were also applied [42]. These consisted of a 10 min formic acid (98%) bath at room temperature, 20 min hydrated autoclaving at 121°C (Prestige Medical, AES Labs, Blackburn Lane, UK) and digestion at 37°C with PK (Roche Diagnostics, Meylan, France) at a concentration of 20 µg/ml for 15 min, with an additional incubation with streptomycin sulfate at 8.75 µg/ml for 1 hour. Endogenous peroxidase activity was also blocked. A peroxidase-labelled avidin-biotin complex (Vectastain Elite ABC, Vector Laboratories, Burlingame, CA) and a solution of diaminobenzidine intensified with nickel chloride (DAB-Ni, Zymed, France) to give black deposits was used to amplify and visualize PrP^d^ binding. The specificity of PrP^d^ immunolabelling was also assessed using uninfected brain sections. Finally, the slides were counterstained with aqueous hematoxylin, dehydrated, mounted using Eukitt and observed under a light microscope BX51 (Olympus, France) coupled to an image analysis workstation (MorphoExpert software, Explora Nova, La Rochelle, France).

## Supporting Information

Figure S1 Vacuolar lesion profiles observed in the brain of C57Bl/6 mice infected with 3 isolates of H-BSE, at second passage, and for one isolate at third passage from a mouse with H-type features or with “C-BSE like” features. Brain vacuolation was scored (means ± standard deviations) on a scale of 0–5 in the following brain areas: 1) dorsal medulla nuclei, 2) cerebellar cortex, 3) superior colliculus, 4) hypothalamus, 5) central thalamus, 6) hippocampus, 7) lateral septal nuclei, 8) cerebral cortex at the level of thalamus, and 9) cerebral cortex at the level of septal nuclei.
(TIF)
