# Supplementary figures and images for: Emergence of Classical BSE Strain Properties during Serial Passages of H-BSE in Wild-Type Mice
Source: PLoS One. 2011 Jan 14;6(1):e15839. doi: 10.1371/journal.pone.0015839 (PMC3021503; doi:10.1371/journal.pone.0015839)

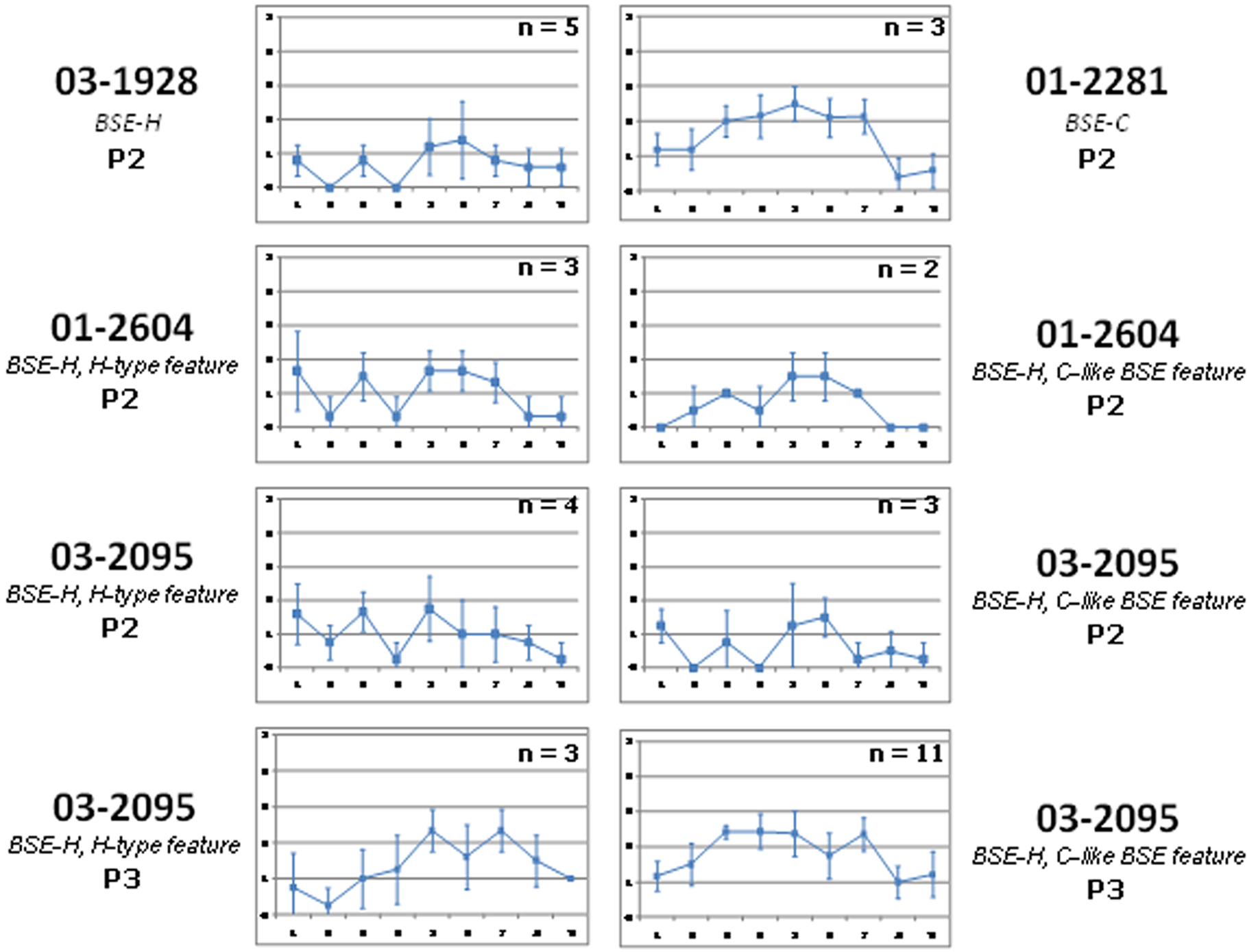

Supplement: Figure S1 — Vacuolar lesion profiles observed in the brain of C57Bl/6 mice infected with 3 isolates of H-BSE, at second passage, and for one isolate at third passage from a mouse with H-type features or with “C-BSE like” features. Brain vacuolation was scored (means ± standard deviations) on a scale of 0–5 in the following brain areas: 1) dorsal medulla nuclei, 2) cerebellar cortex, 3) superior colliculus, 4) hypothalamus, 5) central thalamus, 6) hippocampus, 7) lateral septal nuclei, 8) cerebral cortex at the level of thalamus, and 9) cerebral cortex at the level of septal nuclei. (TIF) [file pone.0015839.s001.tif]
